# Worry and behaviour at the start of the COVID-19 outbreak: Results from three UK surveys (the COVID-19 rapid survey of Adherence to Interventions and responses [CORSAIR] study)

**DOI:** 10.1016/j.pmedr.2021.101686

**Published:** 2021-12-27

**Authors:** Louise E. Smith, Henry W.W. Potts, Richard Amlȏt, Nicola T. Fear, Susan Michie, G. James Rubin

**Affiliations:** a King’s College London, Institute of Psychiatry, Psychology and Neuroscience, UK; b NIHR Health Protection Research Unit in Emergency Preparedness and Response, UK; c University College London, Institute of Health Informatics, UK; d UK Health Security Agency, Behavioural Science and Insights Unit, UK; e King’s Centre for Military Health Research and Academic Department of Military Mental Health, King’s College London, UK; f University College London, Centre for Behaviour Change, UK

**Keywords:** COVID-19, Hand cleansing, Hand washing, Respiratory behaviours, Social distancing, Physical distancing

## Abstract

We aimed to describe worry and uptake of behaviours that prevent the spread of infection (respiratory and hand hygiene, distancing) in the UK at the start of the COVID-19 outbreak (January and February 2020) and to investigate factors associated with worry and adopting protective behaviours. Three cross-sectional online surveys of UK adults (28 to 30 January, n = 2016; 3 to 6 February, n = 2002; 10 to 13 February 2020, n = 2006) were conducted. We used logistic regressions to investigate associations between outcome measures (worry, respiratory and hand hygiene behaviour, distancing behaviour) and explanatory variables. 19.8% of participants (95% CI 18.8% to 20.8%) were very or extremely worried about COVID-19. People from minoritized ethnic groups were particularly likely to feel worried. 39.9% of participants (95% CI 37.7% to 42.0%) had completed one or more hand or respiratory hygiene behaviour more than usual in the last seven days. Uptake was associated with greater worry, perceived effectiveness of individual behaviours, self-efficacy for engaging in them, and having heard more information about COVID-19. 13.7% (95% CI 12.2% to 15.2%) had reduced the number of people they had met. This was associated with greater worry, perceived effectiveness, and self-efficacy. At the start of novel infectious disease outbreaks, communications should emphasise perceived effectiveness of behaviours and the ease with which they can be carried out.

## Highlights

• In Jan-Feb 2020, 20% of the sample were very or extremely worried about COVID-19.
• 40% of participants completed hand or respiratory hygiene behaviours more than usual.
• 14% of the sample reduced the number of people they met before any restrictions.
• Uptake of protective behaviours was associated with greater worry about COVID-19.
• Higher effectiveness and self-efficacy for behaviours were associated with uptake.

## 1 Introduction

The early stages of novel infectious disease outbreaks are usually characterised by uncertainty. Unknowns include basic details about transmissibility, disease severity, risk factors for disease, mode of transmission, and degree of population immunity. In the very early stages of the COVID-19 outbreak, the UK public were exposed to a morass of epidemiological information, disagreements between scientists about the status of the outbreak and its likely future path, frequent admissions of uncertainty from trusted sources, and online confusion, speculation and conspiracy theories. (Stein et al., 2021) In the midst of this, national governments attempted to prepare their citizens for a possible public health crisis and to convey information about behaviours that may help to slow the spread of disease. (Wong et al., 2020)

Uptake of protective behaviours are driven by a more negative appraisal of the threat (greater perceived susceptibility and severity) and a more positive appraisal of the coping response (greater perceived effectiveness and belief that if you wanted to carry out the behaviour, you could [greater perceived self-efficacy]). (Floyd et al., 2000, Han et al., 2016, Rubin et al., 2010) Threat appraisal is likely to be directly linked to the number of infections in one’s locality. In the UK, the first two cases of COVID-19 were declared on 31 January 2020 (Lillie et al., 2020) with seven further cases detected in the subsequent two weeks (see Box 1). During the influenza A H1N1 pandemic, worry was associated with volume of media reporting seen. (Rubin et al., 2010) On 2 February 2020, a public information campaign was launched by the Department of Health and Social Care, England, advising the UK population to adopt respiratory and hand hygiene behaviours. (Department of Health and Social Care. Coronavirus public information campaign launched across the UK [updated 3 February 2020) At the same time, media reports discussed strategies used to prevent transmission in other countries, including restrictions of movement, such as placing regions under “lockdown” measures, (Buckley and Hernández) and reducing contact with others (now known as “physical” or “social distancing”). Trust in the source of information also influences the impact of communications. (Matthews Pillemer et al., 2015, Rubin et al., 2009, Freimuth et al., 2014) At the time of the emergence of COVID-19, politicians were the country’s least trusted profession. (Ipsos, 2019)

**Table:** 

Box 1. Timeline of the start of the COVID-19 outbreak in the UK • 31 January 2020. Two cases detected in the UK; both had recently returned from Hubei province, China [total cases = 2]. • 6 February 2020. One case detected; infection contracted in Singapore [total cases = 3]. • 9 February 2020. One case detected; contact of confirmed UK case, infections contracted in France [total cases = 4]. • 10 February 2020. Four cases detected; contact of confirmed UK case, infections contracted in France [total cases = 8]. • *11 February 2020. World Health Organization names “COVID-19”.* • 12 February 2020. One case detected; infection contracted in China [total cases = 9]. • 23 February 2020. Four cases detected; infections contracted on “Princess Diamond” cruise ship [total cases = 13]. • 27 February 2020. Two cases detected; one infection contracted in Italy, one infection contracted in Tenerife [total cases = 15]. • 28 February 2020. Five cases detected, including first case in Wales and first case in Northern Ireland; two infections contracted in Iran, two infections contracted in Italy (Welsh and Northern Irish infections), one infection contracted in England (first community transmission) [total cases = 20]. • 29 February 2020. Three cases detected; two infections contracted in Italy, one infection contracted in Asia [total cases = 23]. • 1 March 2020. Twelve cases detected; three contacts of confirmed UK case, one infection contracted in England (community transmission), six infections contracted in Italy, two infections contracted in Iran [total cases = 35]. • 5 March 2020. First COVID-19 death in UK announced [total cases = 271]. • *11 March 2020. World Health Organization declares pandemic [total cases = 1,294].* • *16 March 2020. First restrictions imposed in UK [total cases = 3,671].*

Many studies have been published investigating uptake of behaviours that prevent the spread of infection, and factors associated with uptake, at the start of the COVID-19 pandemic (e.g. Atchison et al., 2021, Gibson Miller et al., 2020). However, most of these were conducted as the first set of restrictions were introduced during the first wave of infections (March 2020). Few studies investigated public sentiments and behaviour before this. One study conducted in Italy found that 67% of survey respondents reported washing their hands more often than usual, while 43% cancelled meetings (data collected 24 to 29 February 2020). (Rubaltelli et al., 2020) Another study conducted in Croatia found that women and people without children were more likely to carry out protective behaviours (data collection began 24 February 2020, end date not reported). (Korajlija and Jokic‐Begic, 2020) In the UK, a survey conducted by a market research company showed that 56% of respondents were concerned about COVID-19, 62% were washing their hands with soap and water, and 28% were avoiding large gatherings of people or certain locations (data collected 27 to 29 February 2020). (Brandwatch, 2020) To the best of our knowledge, there are no publicly available data reporting on public sentiment and behaviour in the UK before this date.

In this study, we report data from the first three weekly waves (28 January to 13 February 2020) of a national survey carried out during the COVID-19 outbreak. We assessed population levels of worry, respiratory and hand hygiene behaviours, and reducing the number of people that you met. We investigated associations between worry and sociodemographic characteristics and perceived risk of COVID-19. We investigated associations between self-reported behaviour and sociodemographic characteristics, psychological and contextual factors.

## 2 Method

### 2.1 Design

Weekly online surveys were conducted by BMG research on behalf of the English Department of Health and Social Care (DHSC) (Wave 1: 28 to 30 January 2020, n = 2016; wave 2: 3 to 6 February 2020, n = 2002; wave 3: 10 to 13 February 2020, n = 2006). We analysed these data as part of the CORSAIR study [the COVID-19 Rapid Survey of Adherence to Interventions and Responses study]). (Smith et al., 2021) Standard opinion polling methods (non-probability sampling) were used to aid rapid data collection, which was essential during the evolving crisis.

### 2.2 Participants

Participants were recruited from Respondi, a specialist research panel provider (n = 50,000) and were eligible for the study if they were aged 16 years or over and lived in the UK. Quotas based on age and gender (combined) and Government Office Region reflected targets based on the Office for National Statistics. (Office for National Statistics, 2019) Participants were reimbursed in points (equivalent to approximately 25p) that could be redeemed in cash, gift vouchers or charitable donations.

### 2.3 Study materials

The survey for waves 1 and 2 was developed by DHSC, based on materials developed in 2014 in preparation for a future influenza pandemic by our team. (Simpson et al., 2019) These items were refined in three rounds of qualitative interviews (n = 78) and had their test–retest reliability checked in two telephone surveys (n = 621). (Rubin et al., 2014). Survey materials were substantially expanded in wave 3 (see Appendix A for full items). Unless stated otherwise, we recoded answers of “don’t know” as missing data.

#### 2.3.1 Outcome measures

Participants were asked how worried about COVID-19 they were on a five-point scale (asked in all survey waves). We recoded this item as a binary variable (“not at all”, “not very”, or “somewhat worried” versus “very” or “extremely worried”).

We asked participants if, in the last seven days, they had completed respiratory and hand hygiene behaviours such as washing hands thoroughly and regularly, using hand sanitiser and tissues, and cleaning surfaces “as much as usual,” “more than usual,” “not done this,” or “not applicable” (see Appendix A; wave 3 only). We created a single binary variable indicating whether a participant had completed one or more respiratory or hand hygiene behaviour “more than usual”. For these analyses, answers of “not applicable” were counted as not having completed the behaviour “more than usual”.

Participants were also asked whether they had reduced the number of people they had met in the past seven days (wave 3 only). Answers were recoded to give a single binary variable (reduced the number of people met versus not).

#### 2.3.2 Perceived risk of COVID-19

Participants were asked to what extent they thought COVID-19 posed a risk to themselves and people in the UK (asked in all survey waves).

In wave 3, participants were asked to what extent they agreed that COVID-19 would be a serious illness for them.

#### 2.3.3 Knowledge about COVID-19

In wave 3, participants were asked to what extent they agreed with seven items relating to misinformation that was circulating at the time of data collection (see Appendix A). Individual items were scored from + 2 (strong agreement with a correct answer) to −2 (strong disagreement with a correct answer); we coded “don’t know” as 0. Responses were judged as “true” or “false” based on information provided by the UK Government at the time. Scores were summed and rescaled (range 1 to 29), with higher scores indicating higher knowledge.

#### 2.3.4 Information heard about COVID-19

In wave 3, participants were asked how much they had seen or heard about COVID-19 in the past seven days. Participants were also asked if they had seen or heard the “Catch it, Bin it, Kill it” campaign, (Department of Health and Social Care, 2020) and advice on how to protect themselves and others from COVID-19.

Participants were asked to identify the three sources that they had received most of their information about COVID-19 from in the past seven days from a list of sixteen sources. We created separate binary variables to indicate whether participants had received most of their information from official sources, the mainstream media, or unofficial sources (see Appendix A). For each information source, participants were said to have used that source if they indicated it as one of their top three.

#### 2.3.5 Perceptions about the Government response

Participants were asked to state to what extent they agreed that the Government was putting the right measures in place to protect the British public, they were getting the information they needed, and they knew what to do to limit their risk of contracting COVID-19 (asked in all survey waves). We summed scores to give a single continuous variable indicating satisfaction with the Government response (range 3 to 15, Cronbach’s α = 0.76). Higher scores indicated greater satisfaction.

In wave 3, participants completed an adapted form of the Meyer Credibility Index, focussed on assessing the perceived credibility of Government information about COVID-19. (Meyer, 1988) Scores for individual items were summed (range 4 to 20, Cronbach’s α = 0.76). Lower scores indicated less satisfaction or less credibility.

#### 2.3.6 Effectiveness of, and self-efficacy for, behaviours

Participants were asked to what extent they agreed that individual behaviours were effective at preventing the spread of COVID-19 and how confident they were that they could perform that behaviour (self-efficacy; wave 3 only). We created separate binary variables for perceived effectiveness and self-efficacy for each behaviour (“strongly agree” or “agree” versus “neither agree nor disagree,” “disagree” or “strongly disagree”).

#### 2.3.7 Sociodemographic characteristics

Participants were asked to state: their age at questionnaire completion; gender; whether they had dependent children; whether they themselves or another household member had a chronic illness; their employment status; whether they themselves, a family member, or friend worked for the NHS; and their ethnicity. Index of multiple deprivation was derived from participants’ residential postcode. In wave 3, participants were also asked their highest level of education.

### 2.4 Ethics

This work was conducted as a service evaluation of DHSC’s public communications campaign and was exempt from ethical approval following advice from the King’s College London Psychiatry, Nursing and Midwifery Research Ethics Subcommittee.

### 2.5 Power

A target sample size of 2,000 was used for each wave, allowing a 95% confidence interval of, at most, plus or minus 2.2% for the prevalence estimate for each survey item.

### 2.6 Analysis

Sociodemographic characteristics of participants by wave were compared using χ^2^ tests (categorical data) and one-way ANOVAs (continuous data).

We used binary logistic regressions to calculate univariable associations between worry and sociodemographic characteristics and perceived risk of COVID-19. We used a second set of logistic regressions adjusting for sociodemographic characteristics (excluding education^1^).

We used separate binary logistic regressions to calculate univariable associations between behavioural outcomes (uptake of a respiratory and hand hygiene behaviour, reducing the number of people met) and sociodemographic characteristics, worry about COVID-19, perceived risk of COVID-19, knowledge about COVID-19, information heard about COVID-19, and perceptions about UK Government response. We tested associations between behaviour, effectiveness and self-efficacy separately for each behaviour. We used a second set of logistic regressions adjusting for all sociodemographic characteristics (including education).

For analyses investigating behaviour, we ran *post hoc* logistic regression analyses adjusting for worry about COVID-19 as well as sociodemographic characteristics.

The survey method used quota sampling with weightings. In practice, the weights did not substantially affect rates of worry or uptake of behaviours. Therefore, the analyses reported in this paper are unweighted.

Given the number of analyses conducted on outcomes (worry, n = 16; respiratory and hand hygiene behaviours, n = 26; reducing the number of people met, n = 28), we applied a Bonferroni correction (worry, *p* ≤ 0.003; respiratory and hand hygiene behaviours, *p* ≤ 0.002; reducing the number of people met, *p* ≤ 0.002).

## 3 Results

### 3.1 Participants

Approximately 50% of participants were female (Table 1). There were no significant differences between waves, apart from for age (F (2, 6021) = 3.6, p = .03), with participants being slightly younger in later survey waves.

**Table 1:** Participants’ sociodemographic characteristics by questionnaire wave.

Participant characteristics	Level	Wave of the questionnaire			
		Wave 1(n = 2016)	Wave 2 (n = 2002)	Wave 3 (n = 2006)	p-value
Gender	Male	953 (47.5)	971 (48.8)	986 (49.4)	0.47
	Female	1053 (52.5)	1020 (51.2)	1009 (50.6)	
Age	N, M, SD	N = 2016, M = 48.5, SD = 17.8	N = 2002, M = 48.2, SD = 18.2	N = 2006, M = 48.1, SD = 18.5	0.03*
Dependent children	No	1420 (70.4)	1391 (69.5)	1412 (70.4)	0.76
	Yes	596 (29.6)	611 (30.5)	594 (29.6)	
Chronic illness - self	None	1406 (70.9)	1409 (71.6)	1365 (69.1)	0.22
	Present	577 (29.1)	559 (28.4)	609 (30.9)	
Chronic illness – other household member	None	1740 (87.7)	1699 (86.3)	1681 (85.2)	0.06
	Present	243 (12.3)	269 (13.7)	293 (14.8)	
Employment status	Not working	891 (44.4)	860 (43.3)	897 (45.2)	0.50
	Working	1115 (55.6)	1125 (56.7)	1089 (54.8)	
Work for NHS - self	No	1093 (94.7)	1859 (93.7)	1855 (93.6)	0.28
	Yes	106 (5.3)	124 (6.3)	126 (6.4)	
Work for NHS – members of my family	No	1772 (88.2)	1703 (85.9)	1728 (87.2)	0.09
	Yes	237 (11.8)	280 (14.1)	253 (12.8)	
Work for NHS - friends	No	1796 (89.4)	1791 (90.3)	1792 (90.5)	0.48
	Yes	213 (10.6)	192 (9.7)	189 (9.5)	
Highest educational or professional qualification†	GCSE/vocational/A-level/No formal qualifications	–	–	1350 (67.3)	–
	Degree or higher (Bachelors, Masters, PhD)	–	–	656 (32.7)	–
Index of multiple deprivation	1st quartile (least deprived)	457 (22.7)	436 (21.8)	453 (22.6)	0.92
	2nd quartile	507 (25.1)	486 (24.3)	477 (23.8)	
	3rd quartile	516 (25.6)	535 (26.7)	524 (26.1)	
	4th quartile (most deprived)	536 (26.6)	545 (27.2)	552 (27.5)	
Ethnicity	White	1850 (92.2)	1821 (91.4)	1840 (92.4)	0.43
	Black and minoritized ethnic groups	156 (7.8)	172 (8.6)	151 (7.6)	

*p ≤ 0.05

†Only asked in Wave 3

### 3.2 Worry

Overall, 19.8% of participants (95% CI 18.8% to 20.8%, n = 1191/6024) reported being very or extremely worried about COVID-19. Although rates of worry in wave 2 were significantly lower than waves 1 or 3, this difference was small.

Worry was associated with: greater perceived risk of COVID-19 (to oneself and others in the UK); having dependent children; having a chronic illness (oneself or another household member); being employed; working for the NHS; higher level of deprivation; and belonging to a minoritized ethnic group (Table 2). Having a family member working for the NHS was associated with a lower likelihood of worry. Age was associated with worry in a non-linear manner, with worry declining with increasing age and then flattening.

**Table 2:** Associations between worry about COVID-19 and sociodemographic characteristics and perceived risk of COVID-19.

Participant characteristics	Level	Worry about COVID-19					
		Not at all/not very/somewhat worried n = 4731, n (%)	Very/extremely worried n = 1191, n (%)	Odds ratio (95% CI) for greater worry	p-value	Adjusted odds ratio (95% CI) for greater worry	p-value
Gender	Male	2295 (79.8)	582 (20.2)	Reference	–	Reference	–
	Female	2411 (80.0)	603 (20.0)	0.99 (0.87 to 1.12)	0.83	1.01 (0.88 to 1.16)	0.87
Age	N, M, SD	N = 4731, M = 50.2, SD = 18.0	N = 1191, M = 42.6, SD = 17.7	0.98 (0.97 to 0.98)**	<0.001	0.93 (0.91 to 0.96)**	<0.001
Age: quadratic (age-mean)^2^	–	–	–	–	–	3.64 (2.07 to 6.42)**	<0.001
Dependent children	No	3459 (83.3)	694 (16.7)	Reference	–	Reference	–
	Yes	1272 (71.9)	497 (28.1)	1.95 (1.71 to 2.22)**	<0.001	1.53 (1.31 to 1.79)**	<0.001
Chronic illness – self	None	3271 (79.4	848 (20.6)	Reference	–	Reference	–
	Present	1390 (81.2)	321 (18.8)	0.89 (0.77 to 1.03)	0.11	1.22 (1.04 to 1.43)*	0.02
Chronic illness – other household member	None	4044 (80.3)	994 (19.7)	Reference	–	Reference	–
	Present	617 (77.9)	175 (22.1)	1.15 (0.96 to 1.38)	0.12	1.26 (1.03 to 1.53)*	0.02
Employment status	Not working	2175 (83.8)	419 (16.2)	Reference	–	Reference	–
	Working	2521 (76.7)	765 (23.3)	1.58 (1.38 to 1.80)**	<0.001	1.31 (1.11 to 1.55)**	0.002
Work for NHS – self	No	4468 (80.9)	1052 (19.1)	Reference	–	Reference	–
	Yes	236 (66.3)	120 (33.7)	2.16 (1.72 to 2.72)**	<0.001	1.51 (1.17 to 1.93)**	0.001
Work for NHS – members of my family	No	4081 (79.7)	1037 (20.3)	Reference	–	Reference	–
	Yes	623 (82.2)	135 (17.8)	0.85 (0.70 to 1.04)	0.12	0.79 (0.64 to 0.97)*	0.03
Work for NHS – friends	No	4243 (80.2)	1047 (19.8)	Reference	–	Reference	–
	Yes	461 (78.7)	125 (31.3)	1.10 (0.89 to 1.35)	0.38	0.98 (0.79 to 1.23)	0.88
Highest educational or professional qualification†	GCSE/vocational/A-level/No formal qualifications	1054 (78.9)	282 (21.1)	Reference	–	Reference	–
	Degree or higher (Bachelors, Masters, PhD)	501 (76.7)	152 (23.3)	1.13 (0.91 to 1.42)	0.27	1.00 (0.78 to 1.28)†	0.99
Index of multiple deprivation	1st quartile (least deprived)	1121 (84.5)	205 (15.5)	Reference	–	Reference	–
	2nd quartile	1171 (80.9)	277 (19.1)	1.29 (1.06 to 1.58)*	0.01	1.21 (0.98 to 1.49)	0.07
	3rd quartile	1233 (79.5)	317 (20.5)	1.41 (1.16 to 1.71)**	0.001	1.29 (1.05 to 1.59)*	0.01
	4th quartile (most deprived)	1206 (75.5)	392 (24.5)	1.78 (1.47 to 2.14)**	<0.001	1.49 (1.22 to 1.82)**	<0.001
Ethnicity	White	4442 (82.0)	974 (18.0)	Reference	–	Reference	–
	Minoritised ethnic groups	269 (57.0)	203 (43.0)	3.44 (2.83 to 4.18)**	<0.001	2.50 (2.02 to 3.09)**	<0.001
Questionnaire wave	Wave 1	1557 (79.8)	393 (20.2)	Reference	–	Reference	–
	Wave 2	1619 (81.6)	364 (18.4)	0.89 (0.76 to 1.04)	0.15	0.84 (0.71 to 0.99)*	0.04
	Wave 3	1555 (78.2)	434 (21.8)	1.11 (0.95 to 1.29)	0.20	1.04 (0.88 to 1.23)	0.63
Perceived risk to oneself	5-point Likert-type (1 = no risk at all, 5 = major risk)	N = 4615, M = 2.06, SD = 0.78	N = 1152, M = 3.36, SD = 1.07	4.12 (3.79 to 4.49)**	<0.001	4.06 (3.71 to 4.45)**	<0.001
Perceived risk to people in the UK	5-point Likert-type (1 = no risk at all, 5 = major risk)	N = 4622, M = 2.58, SD = 0.77	N = 1173, M = 3.84, SD = 0.92	4.96 (4.51 to 5.44)**	<0.001	4.87 (4.41 to 5.38)**	<0.001

*p ≤ 0.05

**p ≤ 0.003

†Only asked in Wave 3

†Does not include survey wave as a co-variate as education was only asked about in Wave 3

We conducted a sensitivity analysis using worry as a continuous outcome variable (running multivariable linear regressions). There were only very minor changes to results: being female was additionally associated with worry, while survey wave was not associated.

As *post hoc* analyses, we used independent samples *t*-tests to test whether working for the NHS might be linked to higher knowledge or amount heard about the outbreak. Those who worked for the NHS (n = 126) had lower knowledge about COVID-19 (t(1979) = 5.25, p < .001) than those not working for the NHS (n = 1855). No difference in amount heard about the outbreak was identified.

### 3.3 Respiratory and hand hygiene behaviours

39.9% of participants (95% CI 37.7% to 42.0%, n = 800/2006) indicated that they had completed one or more respiratory or hand hygiene behaviour recommended by the UK Government more than usual in the last seven days. 60.1% of participants (95% CI 58.0% to 62.3%, n = 1206/2006) reported no behaviour change.

Uptake of at least one respiratory or hand hygiene behaviour was associated with: greater worry about COVID-19; having seen or heard information from official sources; having seen recommendations to “Catch it, Bin it, Kill it;” having seen advice on how to protect oneself and others from COVID-19; greater perceived risk from COVID-19 (to oneself and people in the UK); greater perceived severity of COVID-19; greater amount of information heard about COVID-19; having seen or heard information from unofficial sources; poorer knowledge about COVID-19; having a dependent child and working for the NHS (self; Table 3, Table 4). Age was associated with adopting a respiratory or hand hygiene behaviour in a non-linear manner, with behaviour change declining with older age, and then flattening.

**Table 3:** Associations between completing at least one respiratory or hand hygiene behaviour more than usual and sociodemographic characteristics.

Participant characteristics	Level	Respiratory and hand hygiene behaviour					
		Not changed behaviour n = 1206, n (%)	Completed at least one behaviour more than usual n = 800, n (%)	Odds ratio (95% CI) for completing at least one behaviour more than usual	p-value	Adjusted odds ratio (95% CI) for completing at least one behaviour more than usual	p-value
Gender	Male	573 (58.1)	413 (41.9)	Reference	–	Reference	–
	Female	635 (61.9)	384 (38.1)	0.85 (0.71 to 1.02)	0.08	0.86 (0.71 to 1.04)	0.12
Age	N, M, SD	N = 1206, M = 48.92, SD = 17.83	N = 800, M = 46.84, SD = 19.45	0.99 (0.99 to 1.00)*	0.01	0.92 (0.89 to 0.95)**	<0.001
Age: quadratic (age-mean)^2^	–	–	–	–	–	7.45 (3.53 to 15.70)**	<0.001
Dependent children	No	881 (62.4)	531 (37.6)	Reference	–	Reference	–
	Yes	325 (54.7)	269 (45.3)	1.37 (1.13 to 1.67)**	0.001	1.39 (1.11 to 1.74)*	0.004
Chronic illness - self	None	830 (60.8)	535 (39.2)	Reference	–	Reference	–
	Present	360 (59.1)	249 (40.9)	1.07 (0.88 to 1.30)	0.48	1.18 (0.95 to 1.46)	0.14
Chronic illness – other household member	None	1015 (60.4)	666 (39.6)	Reference	–	Reference	–
	Present	175 (59.7)	118 (40.3)	1.03 (0.80 to 1.32)	0.83	1.09 (0.83 to 1.42)	0.55
Employment status	Not working	557 (62.1)	340 (37.9)	Reference	–	Reference	–
	Working	639 (58.7)	450 (41.3)	1.15 (0.96 to 1.38)	0.12	1.23 (0.97 to 1.55)	0.08
Work for NHS – self	No	1138 (61.3)	717 (38.7)	Reference	–	Reference	–
	Yes	53 (42.1)	73 (57.9)	2.19 (1.52 to 3.15)**	<0.001	1.83 (1.24 to 2.70)**	0.002
Work for NHS – members of my family	No	1036 (60.0)	692 (40.0)	Reference	–	Reference	–
	Yes	155 (61.3)	98 (38.7)	0.95 (0.72 to 1.24)	0.69	0.94 (0.71 to 1.25)	0.67
Work for NHS – friends	No	1073 (59.9)	719 (40.1)	Reference	–	Reference	–
	Yes	118 (62.4)	71 (37.6)	0.90 (0.66 to 1.22)	0.49	0.89 (0.64 to 1.23)	0.48
Highest educational or professional qualification	GCSE/vocational/A-level/No formal qualifications	812 (60.1)	538 (39.9)	Reference	–	Reference	–
	Degree or higher (Bachelors, Masters, PhD)	394 (60.1)	262 (39.9)	1.00 (0.83 to 1.21)	0.97	0.94 (0.77 to 1.15)	0.56
Index of multiple deprivation	1st quartile (least deprived)	282 (62.3)	171 (37.7)	Reference	–	Reference	–
	2nd quartile	297 (62.3)	180 (37.7)	1.00 (0.77 to 1.30)	1.00	0.97 (0.74 to 1.28)	0.84
	3rd quartile	301 (57.4)	223 (42.6)	1.22 (0.94 to 1.58)	0.13	1.13 (0.87 to 1.48)	0.36
	4th quartile (most deprived)	326 (59.1)	226 (40.9)	1.14 (0.89 to 1.47)	0.30	1.08 (0.83 to 1.42)	0.56
Ethnicity	White	1123 (61.0)	717 (39.0)	Reference	–	Reference	–
	Black and minoritized ethnic groups	75 (49.5)	76 (50.3)	1.59 (1.14 to 2.21)*	0.01	1.30 (0.91 to 1.87)	0.15

*p ≤ 0.05

**p ≤ 0.002

**Table 4:** Associations between completing at least one respiratory and hand hygiene behaviour more than usual and worry, perceived risk, knowledge about COVID-19, information about COVID-19, and evaluation of the Government response.

	Participant characteristics	Level	Respiratory and hand hygiene behaviour					
			Not changed behaviour n = 1206, n (%)	Completed at least one behaviour more than usual n = 800, n (%)	Odds ratio (95% CI) for completing at least one behaviour more than usual	p-value	Adjusted odds ratio (95% CI) for completing at least one behaviour more than usual	p-value
Worry	Worry	Not at all/not very/somewhat worried	1026 (66.0)	529 (34.0)	Reference	–	Reference	–
		Very/extremely worried	169 (38.9)	265 (61.1)	3.04 (2.44 to 3.79)**	<0.001	2.88 (2.28 to 3.65)**	<0.001
Perceived risk	To oneself	5-point Likert-type (1 = no risk at all, 5 = major risk)	N = 1171, M = 2.26, SD = 0.93	N = 785, M = 2.71, SD = 1.09	1.56 (1.42 to 1.71)**	<0.001	1.51 (1.37 to 1.67)**	<0.001
	To people in the UK	5-point Likert-type (1 = no risk at all, 5 = major risk)	N = 1174, M = 2.79, SD = 0.89	N = 794, M = 3.18, SD = 1.03	1.53 (1.39 to 1.68)**	<0.001	1.51 (1.37 to 1.68)**	<0.001
	Severity of COVID-19 (self)	5-point Likert (1 = strongly disagree, 5 = strongly agree)	N = 1065, M = 3.71, SD = 1.13	N = 748, M = 3.93, SD = 1.03	1.21 (1.11 to 1.32)**	<0.001	1.22 (1.11 to 1.34)**	<0.001
Knowledge	Knowledge	Range 6 to 29	N = 1206, M = 19.69, SD = 3.60	N = 800, M = 18.66, SD = 4.09	0.93 (0.91 to 0.95)**	<0.001	0.94 (0.92 to 0.97)**	<0.001
Information	Amount heard	4-point Likert-type (1 = have not seen or heard anything, 4 = seen or heard a lot)	N = 1198, M = 3.26, SD = 0.74	N = 798, M = 3.39, SD = 0.69	1.28 (1.13 to 1.46)**	<0.001	1.29 (1.13 to 1.48)**	<0.001
	Information source – official sources	No	1005 (63.9)	567 (36.1)	Reference	–	Reference	–
		Yes	201 (46.3)	233 (53.7)	2.05 (1.66 to 2.55)**	<0.001	1.79 (1.42 to 2.26)**	<0.001
	Information source – mainstream media	No	129 (59.2)	89 (40.8)	Reference	–	Reference	–
		Yes	1077 (60.2)	711 (39.8)	0.96 (0.72 to 1.27)	0.76	1.15 (0.84 to 1.58)	0.38
	Information source – unofficial sources	No	804 (62.7)	479 (37.3)	Reference	–	Reference	–
		Yes	402 (55.6)	321 (44.4)	1.34 (1.11 to 1.61)**	0.002	1.29 (1.04 to 1.59)*	0.02
	Advice on protection	No	518 (68.2)	242 (31.8)	Reference	–	Reference	–
		Yes	688 (55.2)	558 (44.8)	1.74 (1.44 to 2.10)**	<0.001	1.69 (1.39 to 2.06)**	<0.001
	Recommendations to “Catch it, Bin it, Kill it”	No	612 (67.0)	301 (33.0)	Reference	–	Reference	–
		Yes	594 (54.3)	499 (45.7)	1.71 (1.42 to 2.05)**	<0.001	1.75 (1.45 to 2.13)**	<0.001
Government response	Satisfaction with government response	Range 3 (lowest) to 15 (highest)	N = 967, M = 10.67, SD = 2.40	N = 727, M = 10.83, SD = 2.44	1.03 (0.99 to 1.07)	0.18	1.03 (0.99 to 1.07)	0.19
	Credibility of government	Range 4 (lowest) to 20 (highest)	N = 836, M = 12.84, SD = 2.45	N = 647, M = 13.3, SD = 2.63	1.00 (0.97 to 1.04)	0.86	1.01 (0.97 to 1.04)	0.76

*p ≤ 0.05

**p ≤ 0.002

We conducted a sensitivity analysis using the number of respiratory and hand hygiene behaviours adopted “more than usual” as a continuous outcome variable (running multivariable linear regressions). There were few changes to results. Additionally associated with behaviour change were being male, being employed, not having friends who worked for the NHS, and living in a more deprived area. Receiving information about COVID-19 from unofficial sources was no longer associated. Where variables were associated with number of respiratory and hand hygiene behaviours adopted, effect sizes were small.

The perceived effectiveness of each behaviour was associated with adopting four of eight individual respiratory and hand hygiene behaviours (see Appendix B). Perceived self-efficacy was associated with adopting four of eight individual respiratory and hand hygiene behaviours.

*Post hoc* analyses investigating uptake of respiratory or hand hygiene behaviours when controlling for worry about COVID-19 did not show meaningful changes in the results for sociodemographic characteristics, or psychological or contextual factors.

### 3.4 Reducing the number of people met

13.7% (95% CI 12.2% to 15.2%, n = 274/2006) people indicated that they had reduced the number of people they had met in the last seven days. 24.4% (95% CI 22.5% to 26.3%, n = 490/2006) had met people as usual; 56.1% (95% CI 53.9% to 58.3%, n = 1125/2006) had not reduced the number of people they had met; and 5.8% (95% CI 4.8% to 6.9%, n = 117/2006) answered “not applicable.”

Reducing the number of people met in the last seven days was associated with: greater worry; greater perceived risk of COVID-19 (to oneself and people in the UK); greater perceived severity of COVID-19; having seen or heard information from official sources; having seen recommendations to “Catch it, Bin it, Kill it”; poorer knowledge about the COVID-19 outbreak; being from a minoritized ethnic group or area of greater deprivation; being male; having dependent children; not having a family member working for the NHS; and not having a friend working for the NHS (Table 5, Table 6). Age was associated with reducing the number of people met in a non-linear manner, with behaviour declining with increasing age (until approximately 60 years old) and then flattening.

**Table 5:** Associations between reducing the number of people you met and sociodemographic characteristics.

Participant characteristics	Level	Reducing the number of people you met					
		Not changed behaviour n = 1732, n (%)	Reduced the number of people you met n = 274, n (%)	Odds ratio (95% CI) for reducing the number of people you met	p-value	Adjusted odds ratio (95% CI) for reducing the number of people you met	p-value
Gender	Male	821 (83.3)	165 (16.7)	Reference	–	Reference	–
	Female	902 (89.4)	107 (10.6)	0.59 (0.45 to 0.77)**	<0.001	0.60 (0.45 to 0.79)**	<0.001
Age	N, M, SD	N = 1732, M = 48.64, SD = 18.45	N = 274, M = 44.63, SD = 18.61	0.99 (0.98 to 1.00)**	0.001	0.95 (0.91 to 1.00)*	0.03
Age: quadratic (age-mean)^2^	–	–	–	–	–	2.78 (0.95 to 8.14)	0.06
Dependent children	No	1242 (88.0)	170 (12.0)	Reference	–	Reference	–
	Yes	490 (82.5)	104 (17.5)	1.55 (1.19 to 2.02)**	0.001	1.41 (1.03 to 1.93)*	0.03
Chronic illness - self	None	1181 (86.5)	184 (13.5)	Reference	–	Reference	–
	Present	525 (86.2)	84 (13.8)	1.03 (0.78 to 1.36)	0.85	1.27 (0.93 to 1.74)	0.13
Chronic illness – other household member	None	1450 (86.3)	231 (13.7)	Reference	–	Reference	–
	Present	256 (87.4)	37 (12.6)	0.91 (0.63 to 1.32)	0.61	0.92 (0.62 to 1.36)	0.67
Employment status	Not working	793 (88.4)	104 (11.6)	Reference	–	Reference	–
	Working	920 (84.5)	169 (15.5)	1.40 (1.08 to 1.82)*	0.01	1.22 (0.87 to 1.72)	0.26
Work for NHS – self	No	1614 (87.0)	241 (13.0)	Reference	–	Reference	–
	Yes	101 (80.2)	25 (19.8)	1.66 (1.05 to 2.62)*	0.03	1.07 (0.65 to 1.77)	0.79
Work for NHS – members of my family	No	1484 (85.9)	244 (14.1)	Reference	–	Reference	–
	Yes	231 (91.3)	22 (8.7)	0.58 (0.37 to 0.92)*	0.02	0.55 (0.34 to 0.89)*	0.01
Work for NHS – friends	No	1536 (85.7)	256 (14.3)	Reference	–	Reference	–
	Yes	179 (94.7)	10 (5.3)	0.34 (0.17 to 0.64)**	0.001	0.29 (0.15 to 0.59)**	0.001
Highest educational or professional qualification	GCSE/vocational/A-level/No formal qualifications	1176 (87.1)	174 (12.9)	Reference	–	Reference	–
	Degree or higher (Bachelors, Masters, PhD)	556 (84.8)	100 (15.2)	1.22 (0.93 to 1.59)	0.15	1.17 (0.88 to 1.58)	0.28
Index of multiple deprivation	1st quartile (least deprived)	407 (89.8)	46 (10.2)	Reference	–	Reference	–
	2nd quartile	425 (89.1)	52 (10.9)	1.08 (0.71 to 1.65)	0.71	0.95 (0.61 to 1.47)	0.80
	3rd quartile	432 (82.4)	92 (17.6)	1.88 (1.29 to 2.75)**	0.001	1.66 (1.12 to 2.47)*	0.01
	4th quartile (most deprived)	468 (84.8)	84 (15.2)	1.59 (1.08 to 2.33)*	0.02	1.41 (0.94 to 2.11)	0.10
Ethnicity	White	1605 (87.2)	235 (12.8)	Reference	–	Reference	–
	Minoritized ethnic groups	115 (76.2)	36 (23.8)	2.14 (1.44 to 3.18)**	<0.001	1.83 (1.18 to 2.83)*	0.01

*p ≤ 0.05

**p ≤ 0.002

**Table 6:** Associations between reducing the number of people you met and worry, perceived risk, knowledge about COVID-19, information about COVID-19 and evaluation of the Government response.

	Participant characteristics	Level	Reducing the number of people you met					
			Not changed behaviour n = 1732, n (%)	Reduced the number of people you met n = 274, n (%)	Odds ratio (95% CI) for reducing the number of people you met	p-value	Adjusted odds ratio (95% CI) for reducing the number of people you met	p-value
Worry	Worry	Not at all/not very/somewhat worried	1414 (90.9)	141 (9.1)	Reference	–	Reference	–
		Very/extremely worried	306 (70.5)	128 (29.5)	4.19 (3.20 to 5.49)**	<0.001	3.76 (2.79 to 5.07)**	<0.001
Perceived risk	To oneself	5-point Likert-type (1 = no risk at all, 5 = major risk)	N = 1685, M = 2.35, SD = 0.97	N = 271, M = 2.96, SD = 1.14	1.70 (1.51 to 1.92)**	<0.001	1.65 (1.45 to 1.88)**	<0.001
	To people in the UK	5-point Likert-type (1 = no risk at all, 5 = major risk)	N = 1696, M = 2.86, SD = 0.93	N = 272, M = 3.49, SD = 1.05	1.88 (1.65 to 2.14)**	<0.001	1.83 (1.59 to 2.11)**	<0.001
	Severity of COVID-19 (self)	5-point Likert (1 = strongly disagree, 5 = strongly agree)	N = 1555, M = 3.77, SD = 1.11	N = 258, M = 4.01, SD = 0.98	1.24 (1.09 to 1.41)**	0.001	1.26 (1.09 to 1.45)**	0.002
Knowledge	Knowledge	Range 6 to 29	N = 1732, M = 19.52, SD = 3.71	N = 274, M = 17.75, SD = 4.28	0.89 (0.86 to 0.92)**	<0.001	0.90 (0.87 to 0.94)**	<0.001
Information	Amount heard	4-point Likert-type (1 = have not seen or heard anything, 4 = seen or heard a lot)	N = 1723, M = 3.31, SD = 0.72	N = 273, M = 3.32, SD = 0.74	1.02 (0.85 to 1.22)	0.83	1.02 (0.84 to 1.23)	0.88
	Information source – official sources	No	1387 (88.2)	185 (11.8)	Reference	–	Reference	–
		Yes	345 (79.5)	89 (20.5)	1.93 (1.46 to 2.56)**	<0.001	1.78 (1.31 to 2.44)**	<0.001
	Information source – mainstream media	No	179 (82.1)	39 (17.9)	Reference	–	Reference	–
		Yes	1553 (86.9)	235 (13.1)	0.69 (0.48 to 1.01)	0.06	0.83 (0.54 to 1.25)	0.37
	Information source – unofficial sources	No	1116 (87.0)	167 (13.0)	Reference	–	Reference	–
		Yes	616 (85.2)	107 (14.8)	1.16 (0.89 to 1.51)	0.26	0.95 (0.70 to 1.28)	0.72
	Advice on protection	No	671 (88.3)	89 (11.7)	Reference	–	Reference	–
		Yes	1061 (85.2)	185 (14.8)	1.31 (1.00 to 1.72)*	0.05	1.29 (0.97 to 1.73)	0.08
	Recommendations to “catch it, bin it, kill it”	No	811 (88.8)	102 (11.2)	Reference	–	Reference	–
		Yes	921 (84.3)	172 (15.7)	1.48 (1.14 to 1.93)*	0.003	1.47 (1.11 to 1.94)*	0.01
Government response	Satisfaction with government response	Range 3 (lowest) to 15 (highest)	N = 1447, M = 10.79, SD = 2.37	N = 247, M = 10.41, SD = 2.65	0.94 (0.89 to 0.99)*	0.02	0.95 (0.89 to 1.00)	0.07
	Credibility of government	Range 4 (lowest) to 20 (highest)	N = 1250, M = 13.00, SD = 2.48	N = 233, M = 13.26, SD = 2.87	0.97 (0.93 to 1.01)	0.16	0.97 (0.92 to 1.02)	0.25
Perceived effectiveness and self-efficacy	Perceived effectiveness	Not effective	912 (94.1)	57 (5.9)	Reference	–	Reference	–
		Effective	738 (77.7)	212 (22.3)	4.60 (3.38 to 6.25)**	<0.001	4.70 (3.38 to 6.55)**	<0.001
	Perceived self-efficacy	Could not carry out behaviour	735 (92.5)	60 (7.5)	Reference	–	Reference	–
		Could carry out behaviour	950 (81.8)	212 (18.2)	2.73 (2.02 to 3.70)**	<0.001	2.95 (2.13 to 4.08)**	<0.001

*p ≤ 0.05

**p ≤ 0.002

In *post hoc* analyses controlling for worry and sociodemographic characteristics, associations between reducing the number of people met and age; having a dependent child; index of multiple deprivation; ethnicity; and perceived severity of COVID-19 for oneself were no longer statistically significant.

## 4 Discussion

The start of all novel infectious disease outbreaks are characterised by uncertainty. Investigating the time period before a major outbreak of infection can help inform planning for future disease outbreaks. Our findings suggest that about 20% of the public reported high levels of worry about COVID-19 at the very start of the outbreak (before community transmission in the UK was confirmed, the pandemic was announced, and any restrictions were introduced). To the best of our knowledge, there are no other publicly available data reporting on this period. Data collected at the end of February indicate that worry increased sharply, with 56% of the population being concerned or very concerned about COVID-19. (Brandwatch, 2020)

Worry was associated with being younger, a parent, having a chronic illness yourself or in your household, being employed, working for the NHS, being from a minoritized ethnic group, and living in a more deprived area of the country. Many of these make intuitive sense, being linked to classic risk factors for more severe illness from respiratory diseases. As the pandemic progressed, these groups were identified as those most at risk of severe disease (e.g. people with specific chronic illnesses and from minoritized ethnic groups); disproportionately affected by restrictions put in place to prevent the spread of infection (e.g. younger people, those living in more deprivation and those with dependent children); and at greater risk of infection (e.g. higher rates of infection in frontline healthcare workers than in the general population). (Gao et al., 2021, Blundell et al., 2020, Office for National Statistics. Coronavirus (COVID-19) Infection Survey pilot: England, 12 June, 2020) Few other studies have investigated predictors of worry about COVID-19. Research conducted in Croatia and Italy has also found that younger age, having a dependent child and people with a chronic health condition had more COVID-19 concerns. (Korajlija and Jokic‐Begic, 2020, Sebri et al., 2021) Unexpectedly, NHS workers had lower knowledge about the outbreak which may have contributed to their higher levels of worry. We are not clear why family members of NHS workers were less worried, but speculate this may be linked to greater access to informal medical advice about their personal risk from COVID-19 or to greater perceived access to healthcare services.

Respiratory and hand hygiene behaviours reduce the spread of acute respiratory infections, (Jefferson et al., 2020) as does reducing physical contact with others. (Ahmed et al., 2018, Fong et al., 2020) Our data indicate that 40% of respondents had completed at least one respiratory or hand hygiene behaviour more than usual in the week that guidance about respiratory and hand hygiene behaviours was introduced in the UK. Approximately 14% of participants reduced the number of people they had met in the previous seven days, although it was not official guidance (until 16 March 2020, Johnson, 2020). One explanation for this may be that people were emulating restrictions imposed in other countries. (Buckley and Hernández) It is likely that in future outbreaks of respiratory viruses, some people may spontaneously adopt respiratory, hand hygiene and physical distancing behaviours.

As in previous outbreaks, and in line with other research carried out at the start of the COVID-19 pandemic, we found that adopting protective behaviours were associated with worry and perceived risk. (Rubin et al., 2009, Jørgensen et al., 2021, Dryhurst et al., 2020) Greater perceived effectiveness of, and self-efficacy for the behaviour were also associated with uptake in our study. (Gibson Miller et al., 2020, Jørgensen et al., 2021, Rogers, 1975, Scholz and Freund, 2021, Seale et al., 2020) Having heard more about COVID-19 was associated with adopting a protective behaviour, similar to other research finding that media exposure was positively correlated with uptake of protective behaviours. (Rubaltelli et al., 2020) This may be mediated by risk perception. (Heydari et al., 2021)

Taken together, results suggest that people who had heard more about the outbreak and who received their information from credible, official sources were more likely to adopt protective behaviours. Preparedness plans for future outbreaks should include a communications campaign that emphasises the effectiveness of protective behaviours and the ease with which behaviours can be completed. Deliberate attempts to increase worry or risk perception to promote uptake of protective behaviours may have unintended negative consequences and should be considered only where levels of risk perception appear disproportionately low and if accompanied by messages emphasising the efficacy of protective behaviours. (Peters et al., 2013)

Analyses of sociodemographic factors associated with adopting protective behaviours before a major outbreak can inform targets of communications for use in future outbreaks. Having completed at least one respiratory or hand hygiene behaviour more than usual was associated with being younger, having a dependent child in your household, and working for the NHS. These associations remained when adjusting for worry. Other studies have also found an association between uptake of preventive behaviours and being a parent. (Korajlija and Jokic‐Begic, 2020) One study conducted in Switzerland found an association between uptake of preventive behaviours and older age (Scholz and Freund, 2021); this has been a common pattern throughout the pandemic. (Smith et al., 2021, Wright et al., 2021) Other studies conducted at the start of the pandemic found no association between age and uptake. (Scholz and Freund, 2021, Seale et al., 2020) For NHS workers and parents, increased uptake of recommended behaviours may have reflected a greater familiarity with, and habitual use of, hygiene behaviours. However, NHS workers were less likely to report having reduced the number of people they had met, as were women. This may have been due to greater occupational contact with people and caring responsibilities in these groups respectively.

Several limitations should be considered for this study. First, behavioural outcomes were self-reported. Social desirability and recall bias may have inflated reported rates of uptake of protective behaviours. However, research suggests that there is no association between social desirability and self-report of health behaviours in online samples. (Crutzen and Goritz, 2010) Whether participants understood the description of the behaviour (e.g. “thorough handwashing”) in the way that we intended is also unclear. Second, while the use of an online market research panel is helpful in ensuring data are collected quickly, people who actively sign up for such panels may not be representative of the general public in terms of, for example, the amount of time they spend online and hence the likelihood of them encountering online public health campaigns. Third, the cross-sectional nature of the data makes it impossible to determine the direction of causality. Fourth, for measures of effectiveness and self-efficacy, we coded answers of “neither agree nor disagree” with “disagree” and “strongly disagree” given that there is evidence suggesting that many people who use middle options in a Likert scale are not expressing the absence of an opinion, but instead using it as a socially desirable way of disagreeing. (Chyung et al., 2017)

## 5 Conclusion

In the early stages of the pandemic in the UK, uptake of protective behaviours was associated with greater worry, risk perceptions, perceived effectiveness of, and self-efficacy for behaviours, and information receipt. All outbreaks of novel infectious diseases start with a period of uncertainty. Our data advance knowledge by giving an important insight into public sentiment in the period before a major outbreak and can be used to inform communications and public health actions at the start of any future outbreak of a novel infectious disease. Preparedness plans should include designing official communications encouraging the uptake of respiratory, hand hygiene and distancing behaviours for use in novel infectious disease outbreaks. Communications should emphasise the effectiveness of these behaviours at preventing the spread of illness and ease with which they can be adopted. Whether worry and uptake of protective behaviours in future novel infectious disease outbreaks will follow the pattern of their predecessor will only be uncovered with time.

## 6 Funding sources

This work was funded by the National Institute for Health Research (NIHR) Health Services and Delivery Research programme (NIHR project reference number 11/46/21) . LS, RA and GJR are supported by the National Institute for Health Research Health Protection Research Unit (NIHR HPRU) in Emergency Preparedness and Response, a partnership between the UK Health Security Agency, King’s College London and the University of East Anglia. RA is also supported by the NIHR HPRU in Behavioural Science and Evaluation, a partnership between the UK Health Security Agency and the University of Bristol. RA is an employee of the UK Health Security Agency. HWWP has received funding from Public Health England and NHS England, consultancy fees to his employer from Ipsos MORI, and has a PhD student who works at and has fees paid by Astra Zeneca. NTF is part funded by a grant from the UK Ministry of Defence. The views expressed are those of the authors and not necessarily those of the NIHR, the UK Health Security Agency, the Department of Health and Social Care or the Ministry of Defence. The Department of Health and Social Care funded data collection (no grant number).

## CRediT authorship contribution statement

**Louise E. Smith:** Conceptualization, Data curation, Formal analysis, Methodology, Writing – original draft. **Henry W.W. Potts:** Conceptualization, Funding acquisition, Methodology, Writing – review & editing. **Richard Amlȏt:** Conceptualization, Funding acquisition, Methodology, Writing – review & editing. **Nicola T. Fear:** Conceptualization, Funding acquisition, Methodology, Writing – review & editing. **Susan Michie:** Conceptualization, Funding acquisition, Methodology, Writing – review & editing. **G. James Rubin:** Conceptualization, Funding acquisition, Methodology, Writing – review & editing.

## Declaration of Competing Interest

All authors had financial support from NIHR for the submitted work; RA is an employee of the UK Health Security Agency; HWWP received additional salary support from Public Health England and NHS England; HWWP receives consultancy fees to his employer from Ipsos MORI and has a PhD student who works at and has fees paid by Astra Zeneca; no other financial relationships with any organisations that might have an interest in the submitted work in the previous three years; no other relationships or activities that could appear to have influenced the submitted work. NTF is a participant of an independent group advising NHS Digital on the release of patient data. All authors are participants of the UK’s Scientific Advisory Group for Emergencies or its subgroups. Preliminary results were made available to DHSC and the UK’s Scientific Advisory Group for Emergencies.

## Footnotes

1 We did not control for education because it was only asked about in wave 3 and was not independently associated with worry about COVID-19.
