# Supplementary material for: Worry and behaviour at the start of the COVID-19 outbreak: Results from three UK surveys (the COVID-19 rapid survey of Adherence to Interventions and responses [CORSAIR] study)
Source: Prev Med Rep. 2021 Dec 27;25:101686. doi: 10.1016/j.pmedr.2021.101686 (PMC8711138; doi:10.1016/j.pmedr.2021.101686)
Supplement: Supplementary data 1 [file mmc1.docx]

Appendix A. Questionnaire materials and top-line results

- Questions 1, 2, 3a-c were asked in all survey waves
- Questions 3d-g, 4 to 9 were only asked in survey wave 3

**Questionnaire**

**The following questions are about the current coronavirus outbreak.**

1. **Overall, how worried are you about coronavirus?**

|  | **Wave 1, n (%)** | **Wave 2, n (%)** | **Wave 3, n (%)** |
| --- | --- | --- | --- |
| Extremely worried | 167 (8.6) | 120 (6.1) | 178 (8.9) |
| Very worried | 226 (11.6) | 244 (12.3) | 256 (12.9) |
| Somewhat worried | 707 (36.3) | 677 (34.1) | 731 (36.8) |
| Not very worried | 617 (31.6) | 676 (34.1) | 607 (30.5) |
| Not at all worried | 233 (11.9) | 266 (13.4) | 217 (10.9) |

Wave 1 base, n=1950 (excluding 66 “don’t know”); Wave 2 base, n=1983 (excluding 19 “don’t know”); Wave 3 base, n=1989 (excluding 17 “don’t know”)

1. **To what extent do you think coronavirus poses a risk to:**
   1. People in the UK?

|  | **Wave 1, n (%)** | **Wave 2, n (%)** | **Wave 3, n (%)** |
| --- | --- | --- | --- |
| Major risk | 125 (6.5) | 101 (5.2) | 163 (8.3) |
| Significant risk | 279 (14.5) | 252 (12.9) | 353 (17.9) |
| Moderate risk | 657 (34.2) | 703 (35.9) | 711 (36.1) |
| Minor risk | 804 (41.9) | 841 (43.0) | 702 (35.7) |
| No risk at all | 55 (2.9) | 61 (3.1) | 39 (2.0) |

Wave 1 base, n=1920 (excluding 96 “don’t know”); Wave 2 base, n=1945 (excluding 57“don’t know”); Wave 3 base, n=1968 (excluding 38 “don’t know”)

- 1. To you personally?

|  | **Wave 1, n (%)** | **Wave 2, n (%)** | **Wave 3, n (%)** |
| --- | --- | --- | --- |
| Major risk | 85 (4.4) | 57 (2.9) | 102 (5.2) |
| Significant risk | 127 (6.6) | 159 (8.2) | 191 (9.8) |
| Moderate risk | 337 (17.6) | 383 (19.7) | 446 (22.8) |
| Minor risk | 966 (50.4) | 974 (50.1) | 939 (48.0) |
| No risk at all | 392 (20.4) | 372 (19.1) | 278 (14.2) |

Wave 1 base, n=1917 (excluding 99 “don’t know”); Wave 2 base, n=1958 (excluding 44 “don’t know”); Wave 3 base, n=1956 (excluding 50 “don’t know”)

1. **To what extent do you agree or disagree with the following statements:**
   1. The Government is putting the right measures in place to protect the British public from coronavirus

|  | **Wave 1, n (%)** | **Wave 2, n (%)** | **Wave 3, n (%)** |
| --- | --- | --- | --- |
| Strongly agree | 167 (10.0) | 240 (13.8) | 254 (14.4) |
| Agree | 707 (42.4) | 876 (50.2) | 889 (50.5) |
| Neither agree nor disagree | 420 (25.1) | 374 (21.4) | 374 (21.3) |
| Disagree | 241 (14.4) | 184 (10.5) | 180 (10.2) |
| Strongly disagree | 91 (5.4) | 71 (4.1) | 62 (3.5) |

Wave 1 base, n=1676 (excluding 340 “don’t know”); Wave 2 base, n=1745 (excluding 257 “don’t know”); Wave 3 base, n=1759 (excluding 247 “don’t know”)

- 1. I feel that I am getting the information I need from the Government and other public authorities on coronavirus

|  | **Wave 1, n (%)** | **Wave 2, n (%)** | **Wave 3, n (%)** |
| --- | --- | --- | --- |
| Strongly agree | 141 (7.6) | 205 (11.0) | 182 (9.7) |
| Agree | 642 (34.4) | 793 (42.4) | 863 (46.1) |
| Neither agree nor disagree | 461 (24.7) | 428 (22.9) | 408 (21.8) |
| Disagree | 446 (23.9) | 314 (16.8) | 307 (16.4) |
| Strongly disagree | 175 (9.4) | 131 (7.0) | 112 (6.0) |

Wave 1 base, n= 1865 (excluding 151 “don’t know”); Wave 2 base, n=1871 (excluding 131 “don’t know”); Wave 3 base, n=1872 (excluding 134 “don’t know”)

- 1. I know what I need to do to limit my risk of contracting coronavirus

|  | **Wave 1, n (%)** | **Wave 2, n (%)** | **Wave 3, n (%)** |
| --- | --- | --- | --- |
| Strongly agree | 227 (12.1) | 284 (15.1) | 319 (16.7) |
| Agree | 751 (40.1) | 892 (47.3) | 971 (51.0) |
| Neither agree nor disagree | 364 (19.5) | 347 (18.4) | 321 (16.9) |
| Disagree | 363 (19.4) | 267 (14.2) | 209 (11.0) |
| Strongly disagree | 166 (8.9) | 94 (5.0) | 85 (4.5) |

Wave 1 base, n=1871 (excluding 145 “don’t know”); Wave 2 base, n=1884 (excluding 118 “don’t know”); Wave 3 base, n=1905 (excluding 101 “don’t know”)

- 1. Information from the Government about coronavirus can be trusted

|  | **Wave 3, n (%)** |
| --- | --- |
| Strongly agree | 200 (11.2) |
| Agree | 868 (48.5) |
| Neither agree nor disagree | 467 (26.1) |
| Disagree | 190 (10.6) |
| Strongly disagree | 66 (3.7) |

Wave 3 base, n=1791 (excluding 215 “don’t know”)

- 1. Information for the Government about coronavirus is accurate

|  | **Wave 3, n (%)** |
| --- | --- |
| Strongly agree | 157 (9.5) |
| Agree | 770 (46.8) |
| Neither agree nor disagree | 492 (29.9) |
| Disagree | 180 (10.9) |
| Strongly disagree | 47 (2.9) |

Wave 3 base, n=1646 (excluding 360 “don’t know”)

- 1. Information from the Government about coronavirus tells the whole story

|  | **Wave 3, n (%)** |
| --- | --- |
| Strongly agree | 125 (7.3) |
| Agree | 500 (29.2) |
| Neither agree nor disagree | 521 (30.5) |
| Disagree | 441 (25.8) |
| Strongly disagree | 123 (7.2) |

Wave 3 base, n=1710 (excluding 296 “don’t know”)

- 1. Information from the Government about coronavirus is biased or one-sided

|  | **Wave 3, n (%)** |
| --- | --- |
| Strongly agree | 103 (6.1) |
| Agree | 361 (21.2) |
| Neither agree nor disagree | 583 (34.3) |
| Disagree | 501 (29.5) |
| Strongly disagree | 152 (8.9) |

Wave 3 base, n=1700 (excluding 306 “don’t know”)

1. **a) How much have you seen or heard about coronavirus in the past 7 days?**

|  | **Wave 3, n (%)** |
| --- | --- |
| I have seen or heard a lot | 914 (45.8) |
| I have seen or heard a fair amount | 817 (40.9) |
| I have seen or heard a little | 242 (12.1) |
| I have not seen or heard anything | 23 (1.2) |

Wave 3 base, n=1996 (excluding 10 “don’t know”)

1. **b) Please tell us for the following options, if you have seen or heard this in the last 7 days…**
   1. Advice on how to protect yourself and others from coronavirus

|  | **Wave 3, n (%)** |
| --- | --- |
| Yes, I have seen or heard this | 1246 (62.1) |
| No, I haven’t seen or heard this | 760 (37.9) |

- 1. Recommendations to “catch it, bin it, kill it”

|  | **Wave 3, n (%)** |
| --- | --- |
| Yes, I have seen or heard this | 1093 (54.5) |
| No, I haven’t seen or heard this | 913 (45.5) |

1. **What three places have you received most of your information about coronavirus from in the past seven days?**

|  | **Wave 3, n (%)** | **Grouping** |
| --- | --- | --- |
| Official helplines (e.g. NHS 111) | 31 (1.5) | Official |
| An NHS website (e.g. NHS.UK) | 172 (8.6) | Official |
| GOV.UK or another Government website | 122 (6.1) | Official |
| National TV news | 1216 (60.6) | Mainstream media |
| Regional TV news | 512 (25.5) | Mainstream media |
| National newspapers (in print) | 353 (17.6) | Mainstream media |
| Regional or local newspapers (in print) | 104 (5.2) | Mainstream media |
| Online news websites (e.g. Guardian, Daily Mail) | 493 (24.6) | Mainstream media |
| Social media sites (e.g. Facebook, Twitter, Instagram) | 461 (23.0) | Social media |
| Search engines (e.g. Google) | 201 (10.0) | Social media |
| National radio | 379 (18.9) | Mainstream media |
| Local radio | 192 (9.6) | Mainstream media |
| Friends/relatives | 241 (12.0) | Social media |
| An NHS GP practice, clinic or hospital | 145 (7.2) | Official |
| Leaflets | 26 (1.3) | Official |
| Posters | 48 (2.4) | Official |
| Other [open end] | 82 (4.1) |  |

(Answer was multi-code, so percentages add to more than 100%, base for all =2006)

1. **For each of the following statements, please tell us to what extent, if at all, you agree or disagree:**
   1. I could catch coronavirus from animals [false]

|  | **Wave 3, n (%)** |
| --- | --- |
| Strongly agree | 154 (10.6) |
| Agree | 403 (27.8) |
| Neither agree nor disagree | 287 (19.8) |
| Disagree | 391 (27.0) |
| Strongly disagree | 214 (14.8) |

Wave 3 base, n=1449 (excluding 557 “don’t know”)

- 1. I could catch coronavirus from packages or products ordered from China [false]

|  | **Wave 3, n (%)** |
| --- | --- |
| Strongly agree | 117 (7.6) |
| Agree | 292 (19.1) |
| Neither agree nor disagree | 293 (19.1) |
| Disagree | 499 (32.6) |
| Strongly disagree | 330 (21.6) |

Wave 3 base, n=1531 (excluding 475 “don’t know”)

- 1. I could catch coronavirus from someone else who has it, even if they do not have any symptoms yet [true]

|  | **Wave 3, n (%)** |
| --- | --- |
| Strongly agree | 809 (44.0) |
| Agree | 842 (45.8) |
| Neither agree nor disagree | 137 (7.4) |
| Disagree | 35 (1.9) |
| Strongly disagree | 17 (0.9) |

Wave 3 base, n=1840 (excluding 166 “don’t know”)

- 1. Coronavirus would be a serious illness for me

|  | **Wave 3, n (%)** |
| --- | --- |
| Strongly agree | 558 (30.8) |
| Agree | 670 (37.0) |
| Neither agree nor disagree | 309 (17.0) |
| Disagree | 220 (12.1) |
| Strongly disagree | 56 (3.1) |

Wave 3 base, n=1813 (excluding 193 “don’t know”)

- 1. It is likely that I have some natural immunity to coronavirus [false]

|  | **Wave 3, n (%)** |
| --- | --- |
| Strongly agree | 77 (5.1) |
| Agree | 270 (18.0) |
| Neither agree nor disagree | 445 (29.7) |
| Disagree | 416 (27.8) |
| Strongly disagree | 290 (19.4) |

Wave 3 base, n=1498 (excluding 508 “don’t know”)

- 1. There is a vaccine available to protect against coronavirus [false]

|  | **Wave 3, n (%)** |
| --- | --- |
| Strongly agree | 64 (4.1) |
| Agree | 117 (7.4) |
| Neither agree nor disagree | 200 (12.7) |
| Disagree | 538 (34.2) |
| Strongly disagree | 652 (41.5) |

Wave 3 base, n=1571 (excluding 435 “don’t know”)

- 1. Antibiotics are an effective treatment for coronavirus [false]

|  | **Wave 3, n (%)** |
| --- | --- |
| Strongly agree | 77 (5.3) |
| Agree | 211 (14.6) |
| Neither agree nor disagree | 296 (20.5) |
| Disagree | 420 (29.1) |
| Strongly disagree | 440 (30.5) |

Wave 3 base, n=1444 (excluding 562 “don’t know”)

- 1. It is currently unsafe to come into contact with someone who has been to Wuhan in China in the past 14 days, regardless of whether they seem ill or well [true]

|  | **Wave 3, n (%)** |
| --- | --- |
| Strongly agree | 890 (47.4) |
| Agree | 706 (37.6) |
| Neither agree nor disagree | 177 (9.4) |
| Disagree | 74 (3.9) |
| Strongly disagree | 30 (1.6) |

Wave 3 base, n=1877 (excluding 129 “don’t know”)

1. **In the past seven days have you…**
   1. Washed your hands thoroughly and regularly with soap and water

|  | **Wave 3, n (%)** |
| --- | --- |
| Done this, same amount as usual | 1362 (67.9) |
| Done this, more than usual | 465 (23.2) |
| Not done this | 147 (7.3) |
| Not applicable | 32 (1.6) |

- 1. Carried tissues with you when out and about

|  | **Wave 3, n (%)** |
| --- | --- |
| Done this, same amount as usual | 1152 (57.4) |
| Done this, more than usual | 300 (15.0) |
| Not done this | 496 (24.7) |
| Not applicable | 58 (2.9) |

- 1. Used tissues when sneezing or coughing

|  | **Wave 3, n (%)** |
| --- | --- |
| Done this, same amount as usual | 1252 (62.4) |
| Done this, more than usual | 301 (15.0) |
| Not done this | 305 (15.2) |
| Not applicable | 148 (7.4) |

- 1. ***If yes to previous question:*** Put tissues in the bin after use

|  | **Wave 3, n (%)** |
| --- | --- |
| Done this, same amount as usual | 1202 (77.4) |
| Done this, more than usual | 269 (17.3) |
| Not done this | 66 (4.2) |
| Not applicable | 16 (1.0) |

Base, n=1553 (excluding 453 not asked)

- 1. Limited the amount you touch your eyes, nose or mouth

|  | **Wave 3, n (%)** |
| --- | --- |
| Done this, same amount as usual | 736 (36.7) |
| Done this, more than usual | 323 (16.1) |
| Not done this | 893 (44.5) |
| Not applicable | 54 (2.7) |

- 1. Cleaned or disinfected surfaces you might touch (such as door knobs or hard surfaces)

|  | **Wave 3, n (%)** |
| --- | --- |
| Done this, same amount as usual | 845 (42.1) |
| Done this, more than usual | 312 (15.6) |
| Not done this | 799 (39.8) |
| Not applicable | 50 (2.5) |

- 1. Carried sanitising hand gel with you when out and about

|  | **Wave 3, n (%)** |
| --- | --- |
| Done this, same amount as usual | 613 (30.6) |
| Done this, more than usual | 280 (14.0) |
| Not done this | 1033 (51.5) |
| Not applicable | 80 (4.0) |

- 1. Used sanitising hand gel to clean your hands

|  | **Wave 3, n (%)** |
| --- | --- |
| Done this, same amount as usual | 814 (40.6) |
| Done this, more than usual | 377 (18.8) |
| Not done this | 751 (37.4) |
| Not applicable | 64 (3.2) |

- 1. Reduced the number of people you meet

|  | **Wave 3, n (%)** |
| --- | --- |
| Done this, same amount as usual | 490 (24.4) |
| Done this, more than usual | 274 (13.7) |
| Not done this | 1125 (56.1) |
| Not applicable | 117 (5.8) |

1. **For each of the following statements, please tell us to what extent, if at all, you agree or disagree:**

An effective way to prevent the spread of coronavirus is to…

- 1. Reduce the number of people you meet

|  | **Wave 3, n (%)** |
| --- | --- |
| Strongly agree | 289 (15.1) |
| Agree | 661 (34.4) |
| Neither agree nor disagree | 613 (31.9) |
| Disagree | 301 (15.7) |
| Strongly disagree | 55 (2.9) |

Wave 3 base, n=1919 (excluding 87 “don’t know”)

- 1. Clean or disinfect surfaces that you might touch (such as door knobs or hard surfaces)

|  | **Wave 3, n (%)** |
| --- | --- |
| Strongly agree | 543 (28.0) |
| Agree | 936 (48.3) |
| Neither agree nor disagree | 363 (18.7) |
| Disagree | 81 (4.2) |
| Strongly disagree | 15 (0.8) |

Wave 3 base, n=1938 (excluding 68 “don’t know”)

- 1. Wash your hands thoroughly and regularly with soap and water

|  | **Wave 3, n (%)** |
| --- | --- |
| Strongly agree | 973 (49.5) |
| Agree | 837 (42.6) |
| Neither agree nor disagree | 119 (6.1) |
| Disagree | 31 (1.6) |
| Strongly disagree | 5 (0.3) |

Wave 3 base, n=1965 (excluding 41 “don’t know”)

- 1. Use sanitising hand gel to clean your hands

|  | **Wave 3, n (%)** |
| --- | --- |
| Strongly agree | 599 (30.9) |
| Agree | 998 (51.4) |
| Neither agree nor disagree | 258 (13.3) |
| Disagree | 73 (3.8) |
| Strongly disagree | 12 (0.6) |

Wave 3 base, n=1940 (excluding 66 “don’t know”)

- 1. Cough or sneeze into tissues, instead of your hands

|  | **Wave 3, n (%)** |
| --- | --- |
| Strongly agree | 924 (47.0) |
| Agree | 844 (43.0) |
| Neither agree nor disagree | 143 (7.3) |
| Disagree | 43 (2.2) |
| Strongly disagree | 11 (0.6) |

Wave 3 base, n=1965 (excluding 41 “don’t know”)

- 1. Put tissues in the bin after you have used them

|  | **Wave 3, n (%)** |
| --- | --- |
| Strongly agree | 894 (45.8) |
| Agree | 867 (44.4) |
| Neither agree nor disagree | 133 (6.8) |
| Disagree | 47 (2.4) |
| Strongly disagree | 13 (0.7) |

Wave 3 base, n=1954 (excluding 52 “don’t know”)

- 1. Limit the amount you touch your eyes, nose or mouth

|  | **Wave 3, n (%)** |
| --- | --- |
| Strongly agree | 473 (24.9) |
| Agree | 850 (44.7) |
| Neither agree nor disagree | 434 (22.8) |
| Disagree | 123 (6.5) |
| Strongly disagree | 20 (1.1) |

Wave 3 base, n=1900 (excluding 106 “don’t know”)

- 1. Keep away from crowded places generally

|  | **Wave 3, n (%)** |
| --- | --- |
| Strongly agree | 378 (19.5) |
| Agree | 890 (46.0) |
| Neither agree nor disagree | 435 (22.5) |
| Disagree | 190 (9.8) |
| Strongly disagree | 43 (2.2) |

Wave 3 base, n=1936 (excluding 70 “don’t know”)

1. **For the following statements, please tell us to what extent, if at all, you agree or disagree:**

How confident are you that, if you wanted to, you could…

1. Reduce the number of people you meet

|  | **Wave 3, n (%)** |
| --- | --- |
| Strongly agree | 429 (21.9) |
| Agree | 733 (37.5) |
| Neither agree nor disagree | 434 (22.2) |
| Disagree | 306 (15.6) |
| Strongly disagree | 55 (2.8) |

Wave 3 base, n=1957 (excluding 49 “don’t know”)

1. Keep surfaces that you might touch clean or disinfected

|  | **Wave 3, n (%)** |
| --- | --- |
| Strongly agree | 714 (36.1) |
| Agree | 942 (47.7) |
| Neither agree nor disagree | 221 (11.2) |
| Disagree | 86 (4.4) |
| Strongly disagree | 13 (0.7) |

Wave 3 base, n=1976 (excluding 30 “don’t know”)

1. Wash your hands thoroughly and regularly with soap and water

|  | **Wave 3, n (%)** |
| --- | --- |
| Strongly agree | 1063 (53.5) |
| Agree | 786 (39.6) |
| Neither agree nor disagree | 110 (5.5) |
| Disagree | 18 (0.9) |
| Strongly disagree | 9 (0.5) |

Wave 3 base, n=1986 (excluding 20 “don’t know”)

1. Carry sanitising hand gel with you when out and about

|  | **Wave 3, n (%)** |
| --- | --- |
| Strongly agree | 765 (38.9) |
| Agree | 815 (41.1) |
| Neither agree nor disagree | 264 (13.4) |
| Disagree | 102 (5.2) |
| Strongly disagree | 23 (1.2) |

Wave 3 base, n=1969 (excluding 37 “don’t know”)

1. Use hand sanitising gel to clean your hands

|  | **Wave 3, n (%)** |
| --- | --- |
| Strongly agree | 810 (41.0) |
| Agree | 892 (45.2) |
| Neither agree nor disagree | 202 (10.2) |
| Disagree | 56 (2.8) |
| Strongly disagree | 14 (0.7) |

Wave 3 base, n=1974 (excluding 32 “don’t know”)

1. Carry tissues with you when out and about

|  | **Wave 3, n (%)** |
| --- | --- |
| Strongly agree | 991 (50.2) |
| Agree | 799 (40.4) |
| Neither agree nor disagree | 146 (7.4) |
| Disagree | 30 (1.5) |
| Strongly disagree | 10 (0.5) |

Wave 3 base, n=1976 (excluding 30 “don’t know”)

1. Put tissues in the bin after you have used them

|  | **Wave 3, n (%)** |
| --- | --- |
| Strongly agree | 1093 (55.4) |
| Agree | 736 (37.3) |
| Neither agree nor disagree | 109 (5.5) |
| Disagree | 24 (1.2) |
| Strongly disagree | 10 (0.5) |

Wave 3 base, n=1972 (excluding 34 “don’t know”)

1. Limit the amount you touch your eyes, nose or mouth

|  | **Wave 3, n (%)** |
| --- | --- |
| Strongly agree | 627 (32.0) |
| Agree | 867 (44.3) |
| Neither agree nor disagree | 322 (16.4) |
| Disagree | 130 (6.6) |
| Strongly disagree | 13 (0.7) |

Wave 3 base, n=1959 (excluding 47 “don’t know”)

Appendix B. Perceived effectiveness of, and self-efficacy for, behaviours

| *Perceived effectiveness of behaviour* |  |  |  |
| --- | --- | --- | --- |
| An effective way to prevent the spread of coronavirus is to… | **Not effective, n (valid %)** | **Effective, n (valid %)** | **Missing, n (total valid)** |
| Clean or disinfect surfaces that you might touch (such as door knobs or hard surfaces) | 459 (23.7) | 1479 (76.3) | 68 (1938) |
| Wash your hands thoroughly and regularly with soap and water | 155 (7.9) | 1810 (92.1) | 41 (1965) |
| Use sanitising hand gel to clean your hands | 343 (17.7) | 1597 (82.3) | 66 (1940) |
| Cough or sneeze into tissues, instead of your hands | 197 (10.0) | 1768 (90.0) | 41 (1965) |
| Put tissues in the bin after you have used them | 193 (9.9) | 1761 (90.1) | 52 (1954) |
| Limit the amount you touch your eyes, nose or mouth | 577 (30.4) | 1323 (69.6) | 106 (1900) |
| Keep away from crowded places generally | 668 (34.5) | 1268 (65.5) | 70 (1936) |
| *Self-efficacy for a behaviour* |  |  |  |
| How confident are you that, if you wanted to, you could… | **Could not carry out behaviour, n (valid %)** | **Could carry out behaviour, n (valid %)** | **Missing, n (total valid)** |
| Keep surfaces that you might touch clean or disinfected | 320 (16.2) | 1656 (83.8) | 30 (2006) |
| Wash your hands thoroughly and regularly with soap and water | 137 (6.9) | 1849 (93.1) | 20 (1986) |
| Carry sanitising hand gel with you when out and about | 389 (19.8) | 1580 (80.2) | 37 (1969) |
| Use hand sanitising gel to clean your hands | 272 (13.8) | 1702 (86.2) | 32 (1974) |
| Carry tissues with you when out and about | 186 (9.4) | 1790 (90.6) | 30 (1976) |
| Put tissues in the bin after you have used them | 143 (7.3) | 1829 (92.7) | 34 (1972) |
| Limit the amount you touch your eyes, nose or mouth | 465 (23.7) | 1494 (76.3) | 47 (1959) |

Table A.1. Table showing frequencies of people stating that individual respiratory and hand hygiene behaviours were effective at preventing the spread of COVID-19, or that they could carry out the behaviour if they wanted.

| Relevant behaviour (In the past seven days have you…) | An effective way to prevent the spread of coronavirus is to… | OR (95%) for completing relevant behaviour more than usual | p-value | aOR (95% CI) for completing relevant behaviour more than usual † | p-value |
| --- | --- | --- | --- | --- | --- |
| Limited the amount you touch your eyes, nose or mouth | Limit the amount you touch your eyes, nose or mouth | 3.12 (2.23 to 4.36)** | <.001 | 3.22 (2.27 to 4.57)** | <.001 |
| Used sanitising hand gel to clean your hands | Use sanitising hand gel to clean your hands | 2.53 (1.73 to 3.70)** | <.001 | 2.78 (1.85 to 4.17)** | <.001 |
| Cleaned or disinfected surfaces you might touch (such as door knobs or hard surfaces) | Clean or disinfect surfaces that you might touch (such as door knobs or hard surfaces) | 2.58 (1.80 to 3.70)** | <.001 | 2.64 (1.81 to 3.87)** | <.001 |
| Put tissues in the bin after use | Put tissues in the bin after you have used them | 1.61 (0.97 to 2.66) | .06 | 1.84 (1.09 to 3.12)* | .02 |
| Carried sanitising hand gel with you when out and about | Use sanitising hand gel to clean your hands | 1.52 (1.05 to 2.21)* | .03 | 1.43 (0.97 to 2.12) | .07 |
| Used tissues when sneezing or coughing | Cough or sneeze into tissues, instead of your hands | 1.14 (0.75 to 1.75) | .53 | 1.36 (0.86 to 2.15) | .18 |
| Washed your hands thoroughly and regularly with soap and water | Wash your hands thoroughly and regularly with soap and water | 1.14 (0.76 to 1.70) | .53 | 1.29 (0.84 to 1.97) | .24 |
| Carried tissues with you when out and about | Cough or sneeze into tissues, instead of your hands | 0.88 (0.59 to 1.30) | .51 | 1.06 (0.69 to 1.62) | .81 |

† Adjusting for all sociodemographic characteristics

**p*≤.05

***p*≤.002

Table A.2. Table showing associations between perceived effectiveness of individual respiratory and hand hygiene behaviours and uptake of individual behaviours

| Behaviour | How confident are you that, if you wanted to, you could… | OR (95%) for completing relevant behaviour more than usual | p-value | aOR (95% CI) for completing relevant behaviour more than usual† | p-value |
| --- | --- | --- | --- | --- | --- |
| Limited the amount you touch your eyes, nose or mouth | Limit the amount you touch your eyes, nose or mouth | 2.69 (1.88 to 3.86)** | <.001 | 2.83 (1.94 to 4.13)** | <.001 |
| Used sanitising hand gel to clean your hands | Use hand sanitising gel to clean your hands | 2.44 (1.60 to 3.72)** | <.001 | 2.69 (1.71 to 4.23)** | <.001 |
| Cleaned or disinfected surfaces you might touch (such as door knobs or hard surfaces) | Keep surfaces that you might touch clean or disinfected | 1.52 (1.05 to 2.19)* | .03 | 1.69 (1.14 to 2.51)* | .01 |
| Carried sanitising hand gel with you when out and about | Carry sanitising hand gel with you when out and about | 1.65 (1.15 to 2.38)* | .01 | 1.51 (1.03 to 2.22)* | .03 |
| Carried tissues with you when out and about | Carry tissues with you when out and about | 1.05 (0.68 to 1.61) | .82 | 1.26 (0.79 to 2.01) | .32 |
| Put tissues in the bin after use | Put tissues in the bin after you have used them | 0.96 (0.59 to 1.58) | .89 | 1.14 (0.67 to 1.91) | .63 |
| Used tissues when sneezing or coughing | Carry tissues with you when out and about | 0.85 (0.57 to 1.27) | .42 | 1.01 (0.66 to 1.56) | .96 |
| Washed your hands thoroughly and regularly with soap and water | Wash your hands thoroughly and regularly with soap and water | 0.64 (0.44 to 0.94)* | .02 | 0.77 (0.51 to 1.15) | .19 |

† Adjusting for all sociodemographic characteristics

**p*≤.05

***p*≤.002

Table A.3. Table showing associations between self-efficacy for individual respiratory and hand hygiene behaviours and uptake of individual behaviours
